# *Aspergillus oryzae* spore germination is enhanced by non-thermal atmospheric pressure plasma

**DOI:** 10.1038/s41598-019-47705-4

**Published:** 2019-08-01

**Authors:** Mayura Veerana, Jun-Sup Lim, Eun-Ha Choi, Gyungsoon Park

**Affiliations:** 1 Plasma Bioscience Research Center, Kwangwoon University, Seoul, 01897 Korea; 2 Department of Plasma Bioscience and Display, Kwangwoon University, Seoul, 01897 Korea; 3 Department of Electrical and Biological Physics, Kwangwoon University, Seoul, 01897 Korea

**Keywords:** Biological physics, Molecular biophysics

## Abstract

Poor and unstable culture growth following isolation presents a technical barrier to the efficient application of beneficial microorganisms in the food industry. Non-thermal atmospheric pressure plasma is an effective tool that could overcome this barrier. The objective of this study was to investigate the potential of plasma to enhance spore germination, the initial step in fungal colonization, using *Aspergillus oryzae*, a beneficial filamentous fungus used in the fermentation industry. Treating fungal spores in background solutions of phosphate buffered saline (PBS) and potato dextrose broth (PDB) with micro dielectric barrier discharge plasma using nitrogen gas for 2 and 5 min, respectively, significantly increased the germination percentage. Spore swelling, the first step in germination, was accelerated following plasma treatment, indicating that plasma may be involved in loosening the spore surface. Plasma treatment depolarized spore membranes, elevated intracellular Ca^2+^ levels, and activated mpkA, a MAP kinase, and the transcription of several germination-associated genes. Our results suggest that plasma enhances fungal spore germination by stimulating spore swelling, depolarizing the cell membrane, and activating calcium and MAPK signaling.

## Introduction

Fermentation with microorganisms is used to produce various compounds in industries related to food, medicine, and bioremediation such as antibiotics, alcohols, amino acids, enzymes, organic acids, and other bioproducts^1–4^. The demand for industrial level fermentation techniques using microorganisms is increasing worldwide, with many countries considering microbial resources to be important assets^5,6^. The Nagoya protocol on access to genetic resources has triggered a worldwide search for effective microbial strains as well as the development of efficient utilization tools^7^. Compared to chemical processes, the use of microorganisms in fermentation has several advantages^5^. Microorganisms are able to multiply fast using a variety of substrates, and microbial reactions are very specific and can be conducted at 25 °C and atmospheric pressure, leading to high production levels. Despite these advantages, certain technical barriers, such as inconsistent survival and stability during cultivation and variable microbial activity, have hindered the efficient application of microorganisms in fermentation. The stable, high density microbial cultivation required for the production of compounds on an industrial scale cannot be achieved under natural culture conditions^8^.

Technologies required for the development of stable and high-density cultures are being actively investigated. For instance, genetic engineering tools are frequently used to generate recombinant strains with improved vitality and functionality^9,10^; however, concerns regarding genetically modified organisms (GMO) and their products have limited the widespread use of this technology. Non-conventional methods such as high pressure, electric fields, and ultrasound have also been applied to improve the yield and productivity of microbial fermentation, yet information regarding these methods is scarce^11^. Non-thermal atmospheric pressure plasma has also been explored as an emerging alternative tool. Plasma is an ionized gas composed of charged and excited particles, reactive neutral species (ROS and RNS), and UV photons^12^. Since non-thermal atmospheric pressure plasma generates various reactive species, a broad spectrum of effects ranging from inactivation to activation may be produced depending on the dosage^13–15^.

Reactive oxygen and nitrogen species (RONS) produced by plasma account for a broad range of effects exerted by plasma. Many studies have demonstrated that plasma-generated RONS play a major role in inactivating microorganisms^16^. Plasma treatment can also stimulate tissue regeneration, cell proliferation, and stem cell differentiation^17–19^, thus plasma-generated RONS may have an important role in these activities. RONS are important signaling molecules that regulate a wide variety of biological and physiological functions, such as cellular growth, gene activation, and the regulation of chemical reactions in living organisms^20–22^. ROS have been shown to regulate conidial germination and germ tube development in *Neurospora crassa* and *Cladosporium fulvum*^23,24^. Moreover, nitric oxide (NO) regulates reproductive processes such as appressorium formation, sporangiophore development, and conidial germination, as well as morphogenesis and pathogenesis in many fungi^25–29^. Additionally, NO regulates nitrogen metabolism during nitrate assimilation in *A*. *nidulans*^30^ and ROS and NO coordinately regulate polar germ tube growth in *Puccinia striiformis* Westend f. sp. *Tritici*^31^.

Enhanced microbial cell growth and differentiation is required for the stable, high-density cultivation of microorganisms, leading to efficient fermentation. Compared to the anti-microbial effect of plasma, the activation of microbial vitality by plasma remains largely unexplored. In the present study, we investigated the role of non-thermal atmospheric pressure plasma in enhancing the initial cell differentiation in the fermenting fungus, *Aspergillus oryzae*, with particular reference to the underlying mechanisms. *A*. *oryzae* is a beneficial filamentous fungus belonging to the phylum Ascomycetes and is widely used to ferment alcohol and soy sauce^32^. It is a generally regarded as safe (GRAS) organism and secretes various hydrolytic enzymes, including alpha-amylase, protease, pectinase, and galactosidase^33,34^. Since the germination of asexual spores (conidia) constitutes the first step in the colonization process, we focused on spore germination to assess the effect of plasma on the enhancement of fungal growth and differentiation.

## Results

### N_2_ plasma may enhance the germination and swelling of *A. oryzae* spores

**Figure 1:**
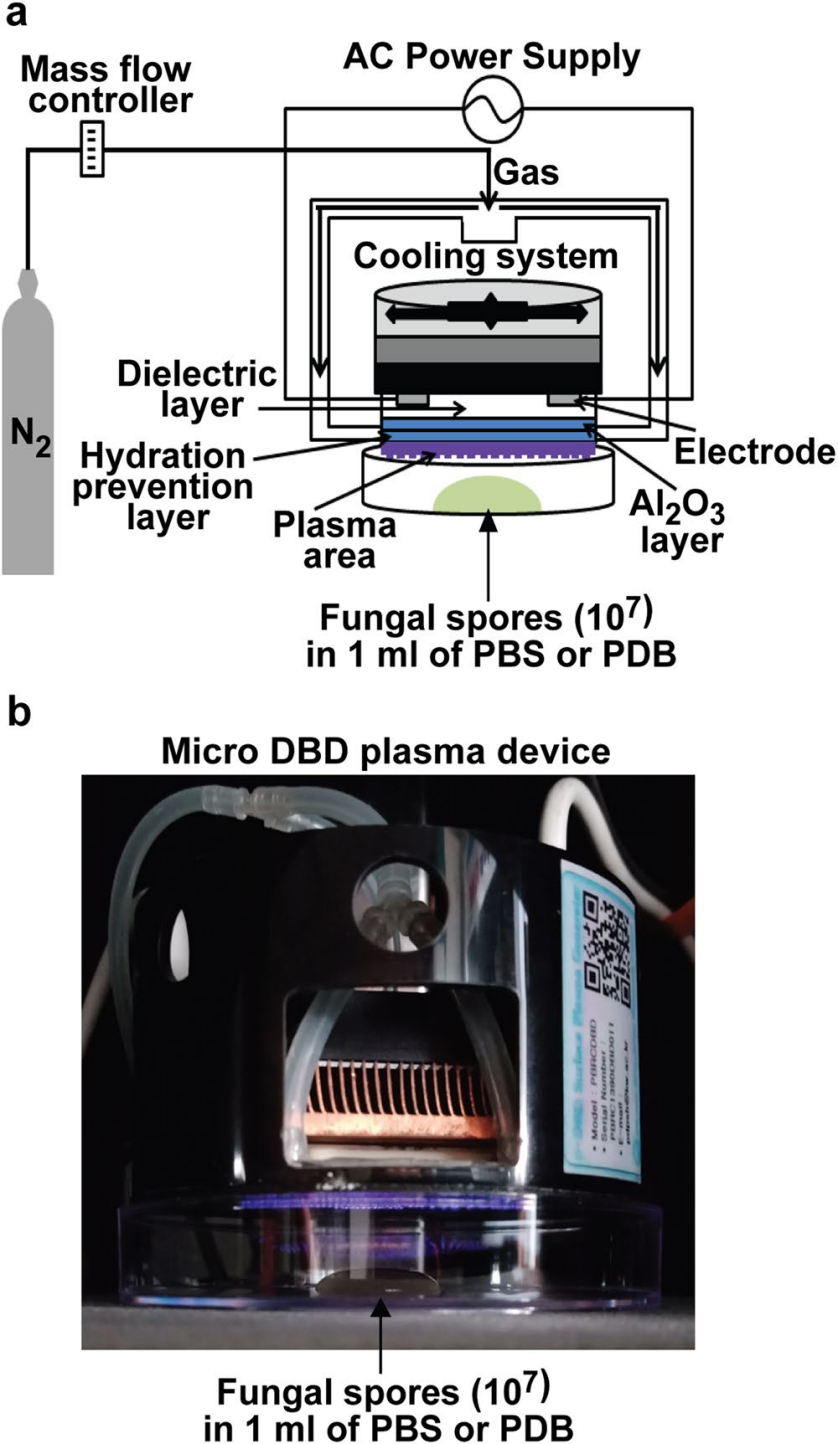
Non-thermal micro DBD plasma device. (**a**) Schematic of the micro DBD plasma device and the experimental set-up for fungal spore treatment with plasma. (**b**) Photograph of spore plasma treatment.

**Figure 2:**
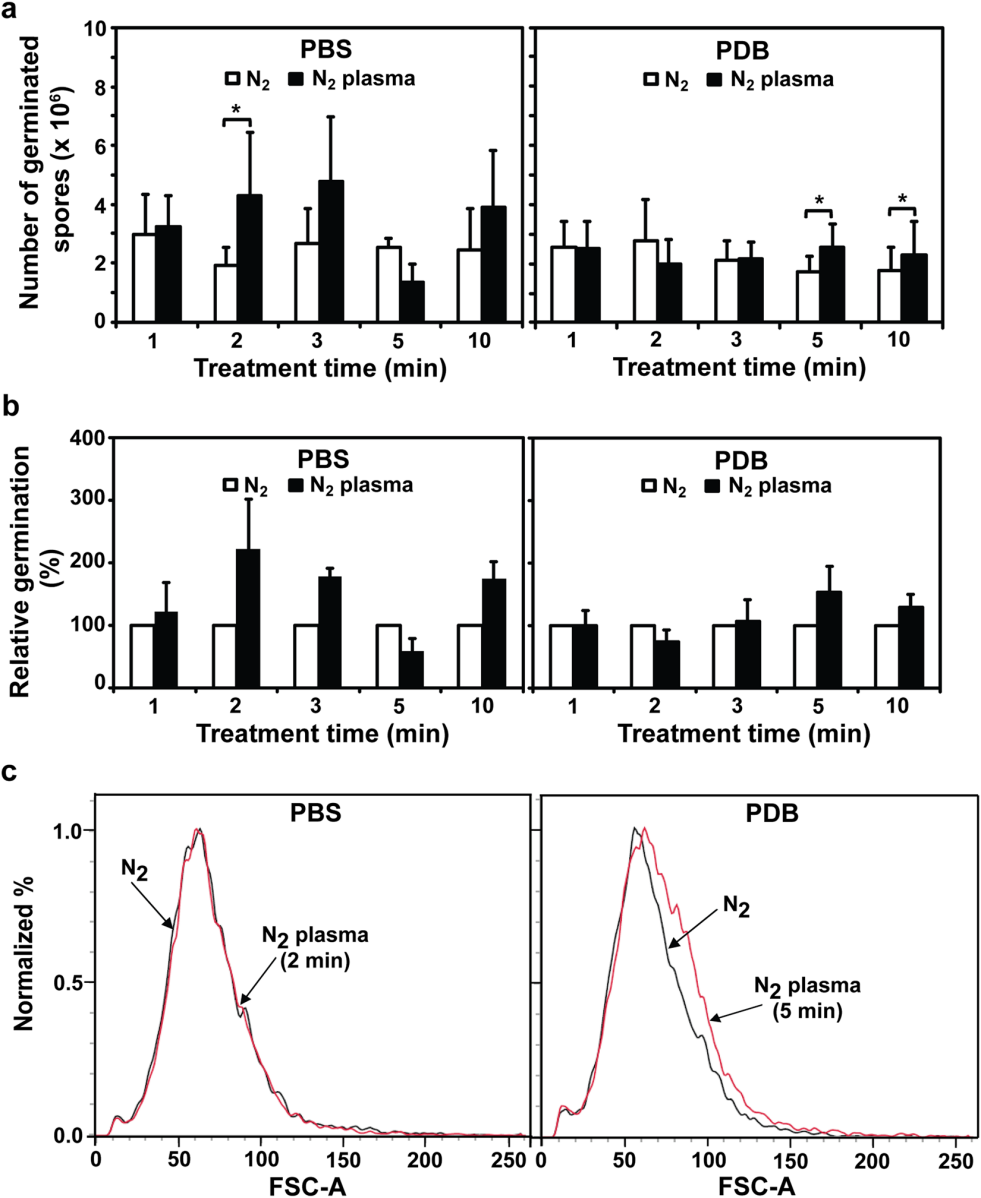
Germination of fungal spores following plasma treatment. (**a**) Number of germinated *A*. *oryzae* spores after treatment with nitrogen (N_2_) gas (control) and plasma in PBS or PDB. (**b**) Relative spore germination percentage following plasma treatment compared to that of the control (N_2_ gas treatment): (number of germinated spores treated with plasma or gas/number of germinated spores treated with gas only) × 100. (**c**) FACS analysis of spore size. Spores treated with plasma in PBS and PDB for 2 min and 5 min, respectively, were analyzed. Black and red lines represent N_2_ gas (control) and plasma treatment, respectively. Each value was averaged from a total of 24 replicates in (**a**,**b**) and FACS analysis was repeated once in (**c**). *p < 0.05.

*A*. *oryzae* spores (10^7^ spores) in two background solutions (PBS and PDB) were treated with nitrogen (N_2_) gas (control) and plasma for 1, 2, 3, 5, and 10 min using a micro DBD plasma device (Fig. 1a,b)^35^. The number of germinated spores was significantly higher (*p* < 0.05) following plasma treatment for 2 min in PBS and for 5 and 10 min in PDB, than in the respective controls (N_2_ gas only; Fig. 2a). Approximately 130–240% relative germination was observed in plasma-treated spores, compared with the control (Fig. 2b). The highest increase in relative germination percentage was observed for the 2 min in PBS and 5 min in PDB treatments (Fig. 2b). Spore germination was enhanced by a shorter plasma treatment time in PBS than in PDB (2 min in PBS vs 5 min in PDB; Fig. 2). The increase in relative spore germination percentage was slowed down in longer plasma treatments in both PBS and PDB (3, 5, 10 min in PBS and 10 min in PDB) (Fig. 2b). Since maximum enhancement in germination was observed for the 2 and 5 min plasma treatments in PBS and PDB, respectively, these treatment conditions were selected for use in further experiments.

Isotropic growth, or spore swelling, is the first stage of spore germination in *A*. *oryzae*^36^. We observed that wild type *A*. *oryzae* spores were swollen after 2 and 4 h of incubation in PBS (Supplementary Fig. S1). To analyze whether plasma enhanced spore swelling during germination, spore size was analyzed following plasma treatment. More plasma-treated spores displayed an increase in size after 1 h than the control in PDB, indicating that more spores were swollen following plasma treatment (Figs 2c and S2). However, no significant difference in spore size was observed between the control and plasma treatment in PBS (Fig. 2c).

### Levels of H_2_O_2_ and NO in plasma-treated solutions were increased

**Figure 3:**
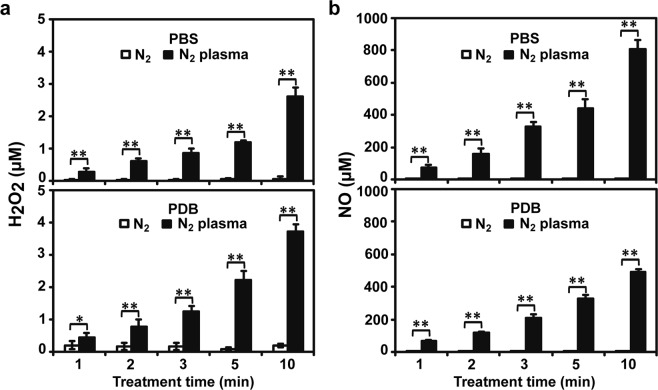
Reactive species levels in background solutions after plasma treatment. (**a**) H_2_O_2_ concentrations in PBS and PDB after treatment with nitrogen (N_2_) gas and plasma. (**b**) NO concentrations in PBS and PDB after treatment with N_2_ gas and plasma. Each value represents the mean of 6–9 replicate measurements. *p < 0.05 and **p < 0.01.

Since fungal spores in the PBS and PDB solutions were treated with plasma, plasma-generated ROS and RNS assimilating into the solutions may affect the fungal spores. Therefore, we measured the levels of ROS and RNS in the PBS and PDB solutions. Due to a lack of assaying methods, only H_2_O_2_ (ROS) and NO (RNS) were quantified in PBS and PDB following plasma treatment. The levels of H_2_O_2_ and NO were found to increase in PBS and PDB following plasma treatment in a time-dependent manner, although only minute amounts were detected in the control (N_2_ gas only) at all time points (*p* < 0.05 or *p* < 0.01) (Fig. 3). H_2_O_2_ concentration increased more rapidly over time in PDB than in PBS, whereas NO concentration increased faster in PBS than in PDB (Fig. 3).

The concentration of NO (approximately 100–800 µM) was much higher than that of H_2_O_2_ (approximately 0.2–4 µM) in both solutions (Fig. 3), indicating that NO may affect fungal spores more than H_2_O_2_. PBS treated with plasma for 2 min exhibited an increase in relative spore germination of over 200%, and approximately 0.6 µM H_2_O_2_ and 160 µM NO were detected (Fig. 3). PDB treated with plasma for 5 min exhibited a 130% increase in relative spore germination and approximately 2.2 µM H_2_O_2_ and 323 µM NO were detected (Fig. 3). Thus, maximum percentage of spore germination was obtained in PBS with lesser H_2_O_2_ and NO than in PDB.

### Spore surface properties after plasma treatment

**Figure 4:**
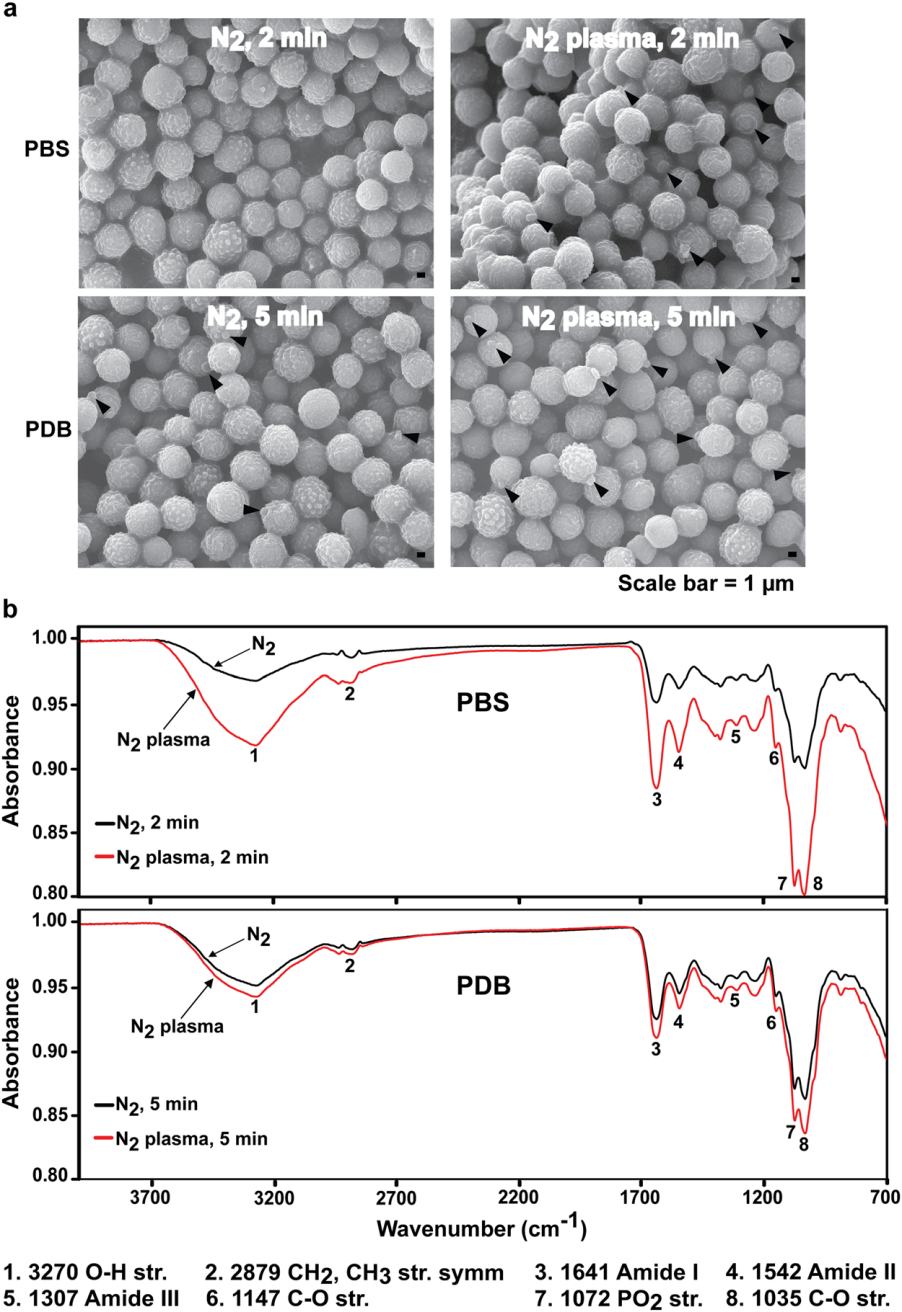
Effect of plasma on spore surface. (**a**) Surface morphology of fungal spores analyzed via SEM after plasma treatment for 2 and 5 min in PBS and PDB solutions, respectively. Arrows indicate the protruded germ tube. (**b**) FTIR spectra of fungal spores following plasma treatment in PBS and PDB for 2 min and 5 min, respectively. Black and red lines represent N_2_ gas and plasma treatments, respectively.

During germination, the cell walls of fungal spores are loosened, leading to spore swelling, with new cell wall and membrane building molecules added to the surface. Thus, the spore surface may be first part of the cell affected by plasma-generated ROS and RNS in PBS and PDB solutions. The spore surface was analyzed using a scanning electron microscope (SEM) and Fourier-transform infrared spectroscopy (FTIR) following plasma treatment. SEM did not indicate a significant difference between the *A*. *oryzae* spore surfaces for the control and plasma treatments, except that germ tube protrusion was observed in more plasma-treated spores than control spores (Fig. 4a).

In FTIR analysis, eight absorption peaks were detected in both the N_2_ gas and plasma-treated spores (Fig. 4b). According to the data of a previous study^37^, these peaks represent functional and chemical groups found in lipids, proteins, and carbohydrates, which are major components of fungal cell walls and membranes. Peak 1 corresponded to the water absorption band at 3350 cm^−1 ^^37^. Although the fungal spores were dried, water molecule bonds were still observed in our FTIR spectra and a water molecule peak was also reported in a previous study^37^. Peaks 2 and 7 were attributed to the functional groups found in phospholipids, while peaks 3–5 may represent protein amide bands, and peaks 6 and 8 corresponded to the chemical groups in carbohydrates^37,38^.

The levels of all eight absorption peaks were higher in the plasma-treated spores than in the control spores (gas treatment) in both PBS and PDB (Fig. 4b). The increase in the peak levels of plasma-treated spores was more intense in PBS than PDB (Fig. 4b). For a more reliable comparison, the levels of absorption peaks 2–8 were normalized based on the level of peak 1 (corresponding to water molecules) since water molecules were present at equal amounts in each sample. After normalization, no significant differences in the relative levels of peaks 2–8 were observed between the control (gas treatment) and plasma treatment in both PBS and PDB (Table 1). However, the relative levels of peaks 7 and 8 were slightly lower after plasma treatment in PBS (Table 1). This may indicate degeneration of the cell membrane and cell wall since peaks 7 and 8 represent the chemical groups in phospholipids and carbohydrates which are major components of the cell wall and membrane.

**Table 1:** Relative level of FTIR absorption peaks.

Peak #	Chemical Group	PBS (2 min)		PDB (5 min)	
		N_2_	N_2_ plasma	N_2_	N_2_ plasma
1	OH stretch	1.000	1.000	1.000	1.000
2	CH_2_, CH_3_ str. symm.	0.434	0.403	0.419	0.421
3	Amide I	1.532	1.408	1.539	1.559
4	Amide II	1.181	1.054	1.117	1.151
5	Amide III	0.975	0.806	0.880	0.908
6	C-O stretch	1.224	1.009	1.155	1.187
7	PO2 stretch	3.000	2.332	2.630	2.674
8	C-O stretch	3.152	2.426	2.808	2.850

### Plasma may induce spore membrane depolarization

To further understand the effect of plasma on the spore surface, the fluidity and membrane potential of the spore cell membranes were analyzed. In particular, the spore membrane is known to become more fluid during fungal spore germination^39^. The fluidity of the spore cell membrane was examined using a lipophilic pyrene dye (AB189819, Abcam, Cambridge, MA, USA). There was no significant difference in the fluidity of the spore cell membrane between the control (N_2_ gas) and plasma-treated spores in either background solution; however, fluidity was slightly higher in the plasma-treated spores in PBS and slightly lower in PDB (Supplementary Fig. S3).

**Figure 5:**
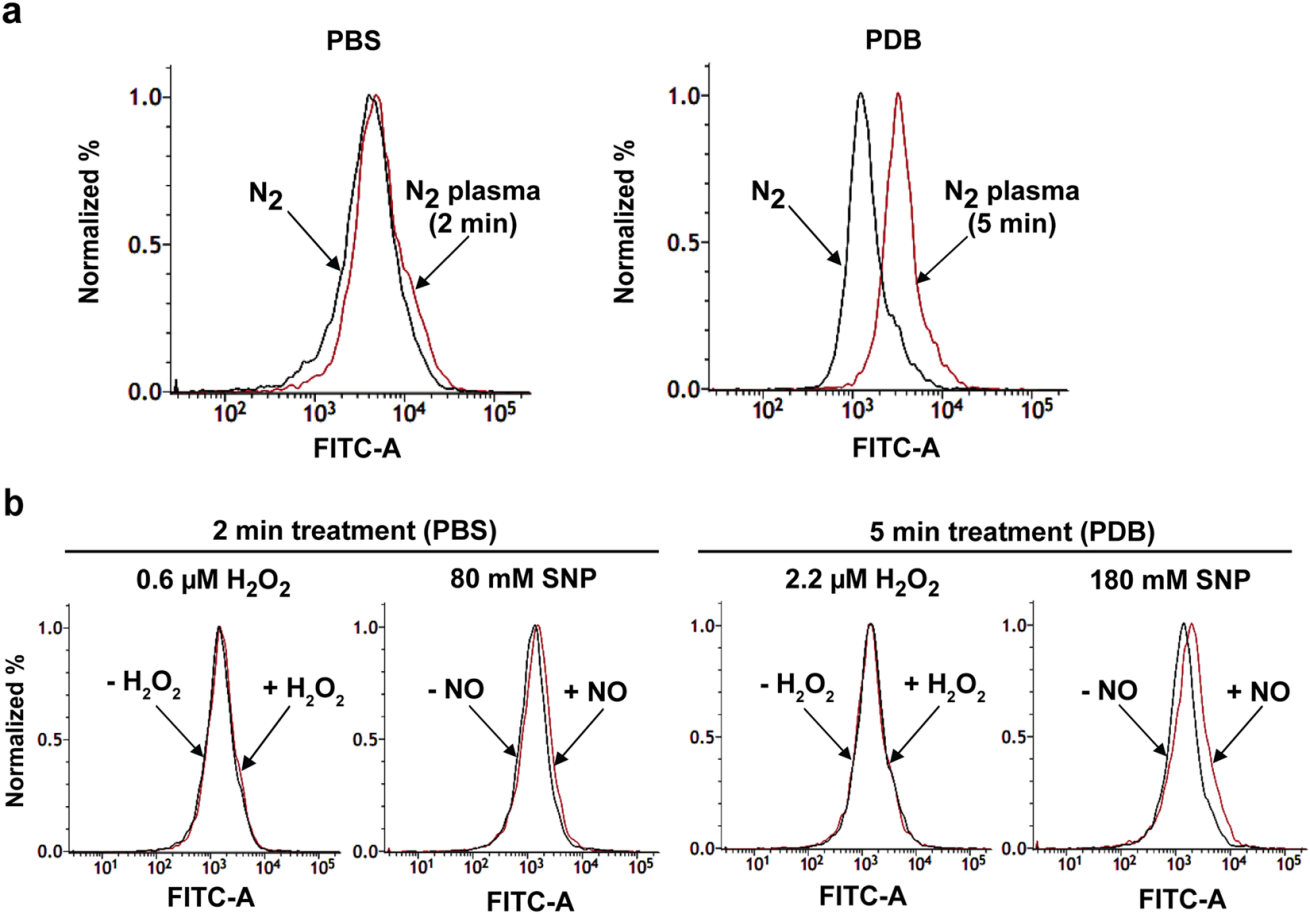
Analysis of *A*. *oryzae* spore membrane potential by DiBAC_4_(3) staining. Histograms show the proportion of fungal spores with different fluorescence intensities following DiBAC_4_ staining (3). Fluorescence indicates that the cell membrane is depolarized. (**a**) The proportion of fungal spores exhibiting different fluorescence intensities following plasma treatment for 2 min in PBS and 5 min in PDB. Black and red lines represent spores treated with nitrogen (N_2_) gas (control) and plasma, respectively. (**b**) The proportion of fungal spores exhibiting different fluorescence intensities following treatment with the indicated concentrations of SNP or H_2_O_2_ dissolved in PBS and PDB. Treatment time was 2 min for PBS and 5 min for PDB solutions. Black and red lines represent spores treated without and with H_2_O_2_ and SNP, respectively.

Spore membrane potential was analyzed by staining spores with a voltage sensitive dye DiBAC_4_(3) (ThermoFisher Scientific, Waltham, MA, USA), revealing that the fluorescence intensity of the plasma-treated spores was higher than that of the control in both PBS and PDB, with a more pronounced increase in PDB than in PBS (Fig. 5a). This indicated that spore membrane depolarization was increased following plasma treatment in both PBS and PDB. Since we detected H_2_O_2_ and NO in the plasma-treated PBS and PDB, we examined the importance of these reactive species in membrane depolarization. We directly exposed fungal spores to H_2_O_2_ and NO dissolved in PBS and PDB, with the concentration of each species adjusted to the same level as measured in plasma-treated PBS (2 min) and PDB (5 min). For NO treatment, the NO donor sodium nitroprusside (SNP) was used at concentrations of 80 and 180 mM in PBS and PDB, respectively. These concentrations of SNP produced the same level of NO measured in PBS and PDB treated with plasma for 2 and 5 min, respectively (80 mM; 164.9 µM NO, 180 mM; 339.8 µM NO; Supplementary Fig. S4). When fungal spores were incubated in PBS containing H_2_O_2_ (0.6 µM) for 2 min or in PDB containing H_2_O_2_ (2.2 µM) for 5 min, no clear change in fluorescence intensity was observed between the control (without H_2_O_2_) and H_2_O_2_ solutions (Fig. 5b); however, the fluorescence intensity of the SNP-treated spores was higher and more pronounced in PDB than in PBS (Fig. 5b). Thus, compared with H_2_O_2_, NO may play a major role in the depolarization of spore membranes.

### Plasma treatment increases intracellular Ca^2+^ levels

**Figure 6:**
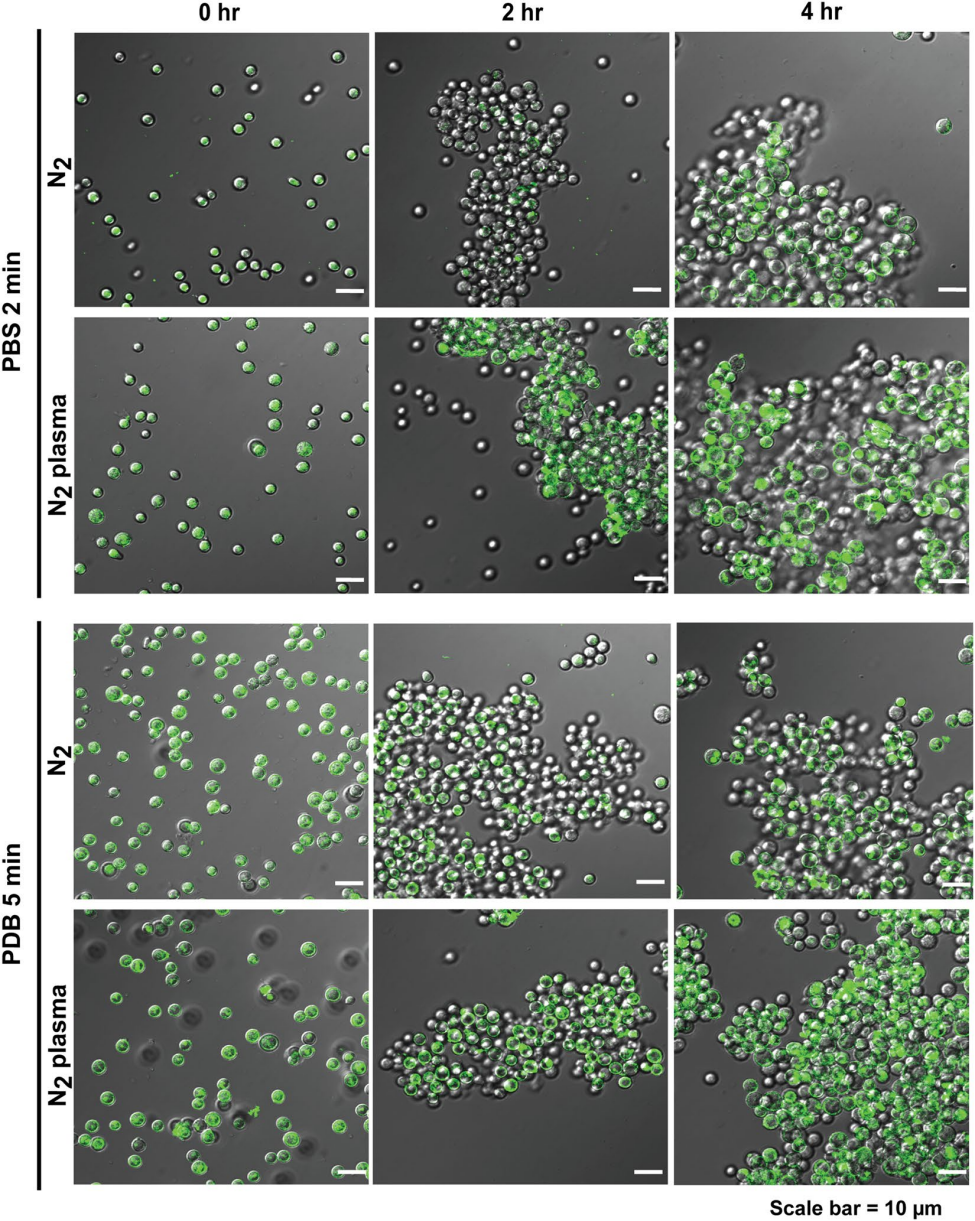
Intracellular Ca^2+^ levels in fungal spores following plasma treatment. Fungal spores labeled with the Fluo-3-AM probe examined under a confocal laser scanning microscope at laser 488 nm. Fungal spores were treated with nitrogen gas and plasma for 2 min in PBS and 5 min in PDB solutions and then incubated at 30 °C for 0, 2, and 4 h.

Since membrane depolarization indicates the activation of ion influx systems such as Ca^2+^ ion channel, we examined the change in intracellular Ca^2+^ levels following plasma treatment using the fluorescent Ca^2+^ probe, fluo-3 AM. In both PBS and PDB, the number of plasma-treated spores exhibiting fluorescence (intracellular Ca^2+^) was higher than that of the control spores during incubation for 2 and 4 h, indicating that plasma treatment triggered an increase in the intracellular Ca^2+^ level (Figs 6 and S5). In most spores, intracellular Ca^2+^ fluorescence was largely observed at 0 h, and subsequently on the cell surface as well as in the cell interior following 2 and 4 h incubation (Figs 6 and S5). Thus, the process appears to be accelerated in plasma-treated spores. Fluorescence was initially observed on the cell surface of plasma-treated spores after 2 h incubation in both PBS and PDB, whereas fluorescence was mostly observed inside the cells of control spores (gas treated) (Figs 6 and S5). More plasma-treated spores exhibited cell surface and intracellular fluorescence following a 4 h incubation period than the control spores (Figs 6 and S5). Intracellular fluorescence appeared to be located mostly in vacuole-like structures (an intracellular Ca^2+^ ion reservoir) after a longer incubation period (2 and 4 h). The intensity as well as the area of fluorescence was greater in plasma-treated spores than in the control spores (Figs 6 and S5), indicating that intracellular Ca^2+^ ion levels may be enhanced by nitrogen plasma, likely through the activation of Ca^2+^ ion channels, and this may be associated with the promotion of spore germination in *A*. *oryzae*.

### Plasma treatment promotes MAP kinase phosphorylation and germination-associated gene expression

**Figure 7:**
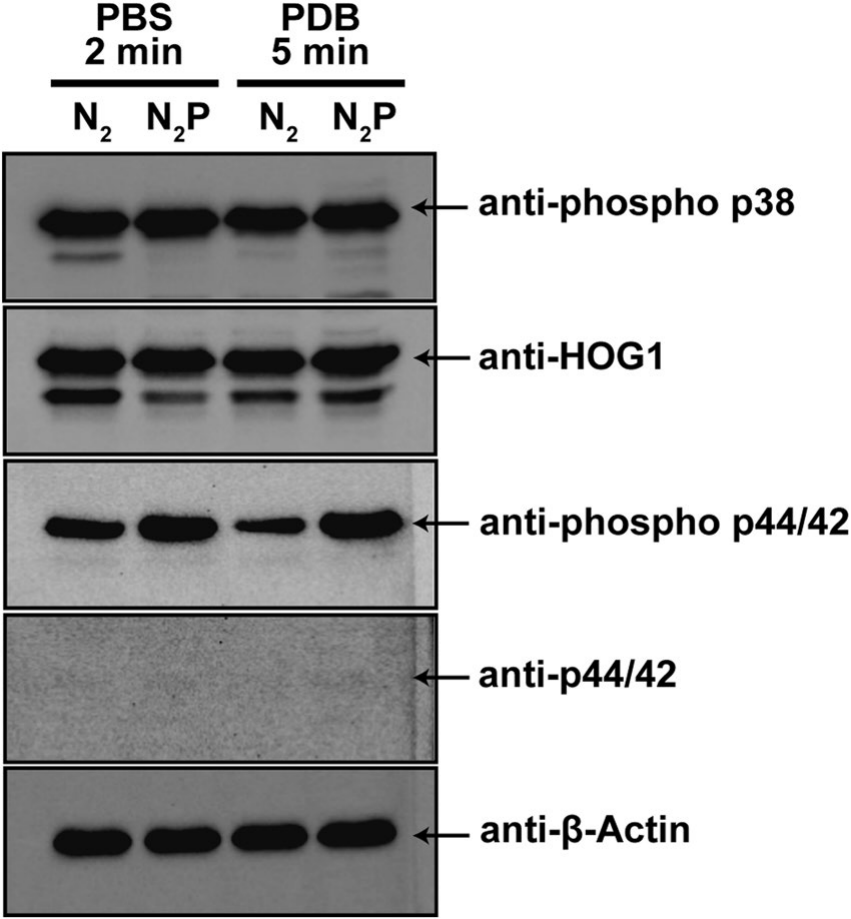
Western blot analysis of MAPK phosphorylation following plasma treatment. Total proteins were extracted from fungal spores incubated for 4 h following plasma treatment (2 min in PBS and 5 min in PDB). N_2_; treatment with only N_2_ gas, N_2_P; treatment with N_2_ and plasma. Arrows indicate protein bands corresponding to each MAP kinase (hogA or mpkA) with or without phosphorylation and β-actin (reference protein). The phosphorylation and protein levels of each MAP kinase were detected using following antibodies: hogA (expected molecular weight 41.8 kDa); anti-phospho p38 (phosphorylation) and anti-Hog1 (protein), mpkA (expected molecular weight 47.8 kDa); anti-phospho p44/42 (phosphorylation) and anti-p44/42 (protein).

Since intracellular Ca^2+^ ions are secondary messengers, they may activate cellular signaling pathways associated with spore germination. In addition, mitogen-activated protein kinases (MAPKs), the most important evolutionarily conserved cellular signaling components in eukaryotic organisms, are known to play a role in *A*. *oryzae* spore germination. The activation of MAPKs in *A*. *oryzae* was examined by assessing the level of kinase phosphorylation (activation) 4 h after plasma treatment. Western blot analysis demonstrated that the level of phosphorylated mpkA, a MAPK homologous to Slt2p in yeast (detected using an anti-phospho p44/42 antibody), was higher in plasma-treated spores in both PBS and PDB than in the control spores (Figs 7 and S6). However, the phosphorylation levels of a MAPK homologous to Hog1p in yeast (hogA, detected using an anti-phospho p38 antibody) was not significantly different in the plasma-treated and control spores in both PBS and PDB (Figs 7 and S6). No significant difference in the protein level of MAPKs was observed between the plasma-treated spores and the control spores in both PBS and PDB (Figs 7 and S6). These results indicate that a plasma-induced increase in intracellular Ca^2+^ levels may activate mpkA.

Since calcium and MAPK signaling may eventually activate the expression of effector genes, we analyzed the transcription levels of 10 putative germination-associated genes (Table 2). Among these genes, nine are known to be transcribed more than 20-fold in conidia during the early stages of germination and one (AtfA) is a transcription factor known to activate *A*. *oryzae* spore germination^40,41^. The transcription levels of these 10 genes were monitored for up to 4 h following plasma treatment since germ tube protrusions (early stage of germination) occur only after 4 h (Supplementary Fig. S1).

**Table 2:** List of primers used in qPCR for 10 putative genes related to spore germination and a reference gene (18S ribosomal RNA).

*A*. *oryzae*r (Gene ID number)	Putative gene function	Primer sequences
AO090005001280*	Extracellular thaumatin domain protein, putative	Forward-TCCCTCTGTCAACTGTGCTG
		Reverse-TGCTGACACTGAAGGAGGTG
AO090011000630	Carbamoyl-phosphate synthase, large subunit	Forward-TCGAATGGTAAGCTCCTGCT
		Reverse-TCAGCAATCTCCTTGACACG
AO090011000649	Purine-cytosine permease	Forward-CGTTTTGGCCTGTATTGGTT
		Reverse-TAGCACACCTGCAAATCCTG
AO090005000164	Conserved hypothetical protein	Forward-CCATCTCAACGTCTCAAGCA
		Reverse-CCAACGACAAGAAGTGAGCA
AO090011000497	Hypothetical nuclear protein	Forward-TACACCGATGGAGGGAGTTC
		Reverse-AGAGGGTTACTGCTGCCTGA
AO090001000546	5′-methylthioadenosine phosphorylase	Forward-AGGCTACCGCAGATGTCACT
		Reverse-CGAACTTGACAGAGCCTTCC
AO090026000687	MYB DNA binding protein (Tbf1), putative	Forward-ACATCATCAGGAAGGCCAAC
		Reverse-GGCAATGAAAGCCTGTGTTT
AO090003000611	Hydroxymethylglutaryl-CoA synthase	Forward-TGCTCGTCCTCAGAACATTG
		Reverse-TGTCCCAGACCGATTGTGTA
AO090003001496	Conserved hypothetical protein	Forward-CCACTGGCCTCACTACCATT
		Reverse-TACAGGTTGAGGGCCAAGAC
AO090003000685	ATF/CREB family transcription factor, AtfA	Forward-GTCGCCAGAGGAAGAAACAG
		Reverse- AGAAACCGGGCAGTCCTTAT
AO090206r00001	18S ribosomal RNA	Forward-GGAAACTCACCAGGTCCAGA
		Reverse-AGCCGATAGTCCCCCTAAGA

^*^Ten putative genes related to *A*. *oryzae* spore germination were selected for qPCR based on the report by Hagiwara *et al*.^41^; genes with more than a 20-fold fragments per kilobase of transcript per million mapped reads (FPKM) ratio in 1 h-grown conidia compared with conidia, as detected by RNA-sequencing analysis.

**Figure 8:**
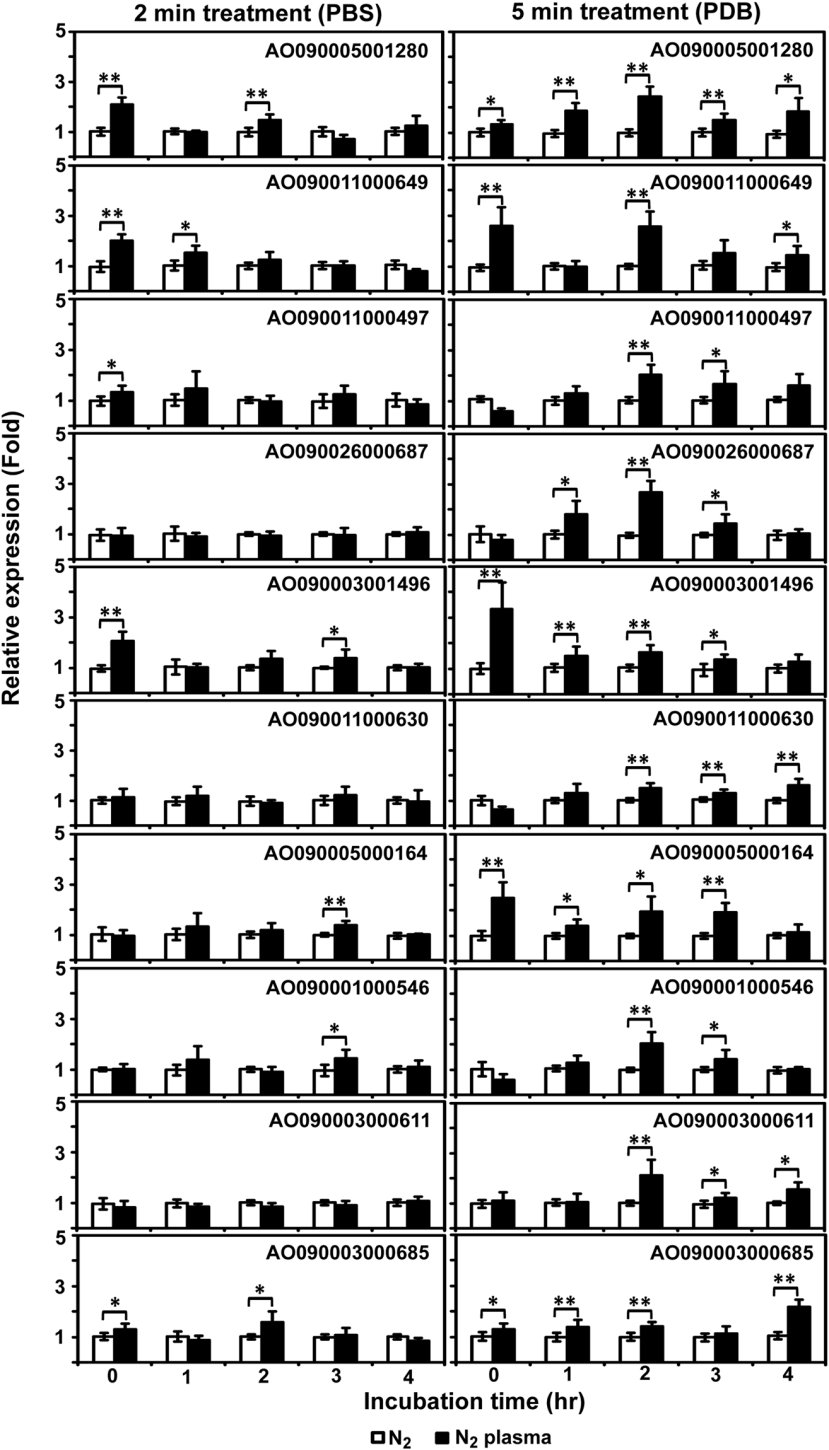
Expression of germination-related genes following plasma treatment in PBS and PDB. The mRNA levels of 10 germination-related genes were quantified using QPCR. Spores were treated with nitrogen plasma for 2 min in PBS and 5 min in PDB and then incubated for 0, 1, 2, 3, and 4 h. Each value is the mean of 3–9 replicate measurements. *p < 0.05, **p < 0.01.

The mRNA expression of six genes (including AtfA) was significantly increased immediately after nitrogen plasma treatment (0 h) in PBS and PDB (*p* < 0.05 or *p* < 0.01, Fig. 8). Among these, the expression of three genes encoding an extracellular thaumatin domain protein homolog (AO090005001280), a purine-cytosine transporter homolog (AO090011000649), and a conserved hypothetical protein (AO090003001496), was significantly higher in both PBS and PDB at 0 h following plasma treatment compared with the control (gas treatment; Fig. 8, *p* < 0.01). In PDB, the expression levels of all 10 genes were maintained relatively highly following plasma treatment throughout the incubation period (0–4 h; Fig. 8). The expression of AO090003000685 (AtfA) was significantly higher in the plasma-treated spores in both PBS and PDB at 0 h, and this transcription level was maintained throughout the incubation period in PDB (*p* < 0.05 or *p* < 0.01, Fig. 8). AO090005001280, an extracellular thaumatin domain protein detected on swollen conidial surfaces and highly expressed during the early stages of germination, was transcribed more following plasma treatment throughout the incubation period (Fig. 8).

## Discussion

Our results clearly demonstrated that micro DBD plasma may accelerate the germination (germ tube development) of *A*. *oryzae* spores. Since spore germination is the initial step of fungal cell differentiation and growth, the activation of germination in this way may lead to the successful colonization of beneficial fungi in artificial culture systems. The activation effects of plasma, particularly on cell proliferation and differentiation, have been reported in animal and human cells, such as mesoderm-derived human adult stem cells, osteoblastic precursor cells, and adipose tissue derived stem cells^42,43^; however, there is a lack of similar information in microbial cells. Most studies have focused on the plasma-induced inactivation of microorganisms harmful to human life^44^. Our study indicates that there is a certain plasma dose window that activates microorganisms. A recent study supporting this notion demonstrated that the multiplication and motility of a bacteria beneficial to plant growth can be enhanced by plasma treatment^35^. The activation of fungal cells by plasma has not yet been reported; therefore, to the best of our knowledge, this is the first study to provide experimental evidence demonstrating the activation of fungal cell differentiation by plasma. Plasma is likely to have a potential for enhancing the biological activity in both bacterial and fungal cells.

In our study, the plasma treatment times associated with an optimal increase in spore germination were different for PBS (2 min) and PDB (5 min) solutions, although longer treatment times eventually inhibited spore germination in both PBS and PDB. These results indicate that the effect of plasma on fungal spores may be affected by the environment surrounding the spores. The pH of PDB (~5) was generally lower than that of PBS (~7); however, no significant change in pH was observed after plasma treatment (Supplementary Fig. S7). The difference in the pH of PBS and PDB may affect the interactions between plasma-generated reactive species and fungal spores; however, our study provides no evidence supporting this hypothesis. Another possibility is that reactions between plasma-generated ROS and RNS and the chemicals in PDB may reduce the levels of reactive species, thus longer treatment times may be required to achieve the same effect. Since potato extract is a major component in PDB and the antioxidant compounds such as carotenoids and phenolic compounds are present in potato peel and flesh^45^, plasma generated ROS and RNS are likely to be scavenged by antioxidants in PDB.

**Figure 9:**
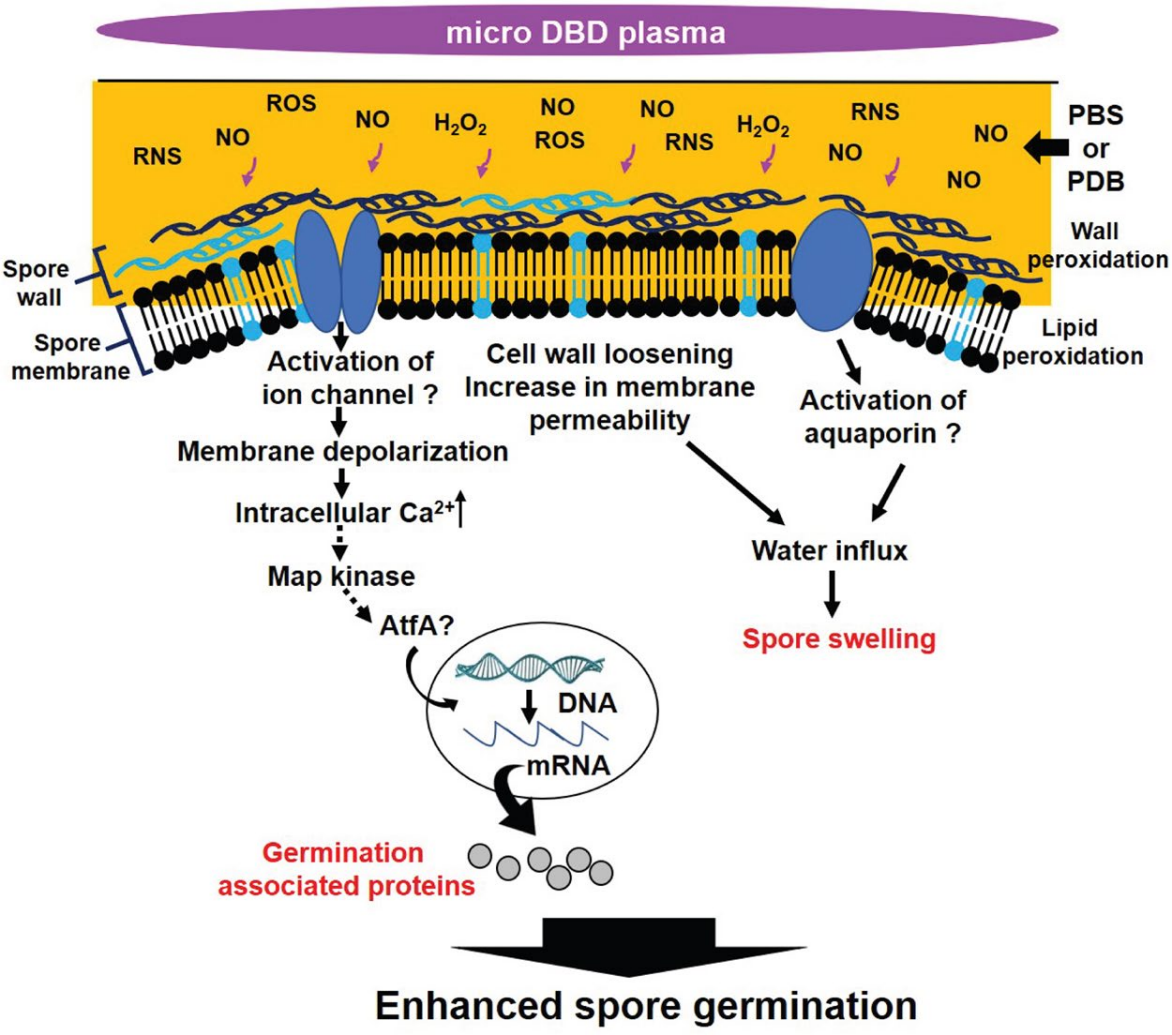
Proposed model for the mechanism(s) of plasma-activated spore germination.

Our findings suggest several possibilities regarding the mechanism(s) underlying the activation of spore germination by plasma (Fig. 9): (1) plasma-generated reactive species may enhance spore swelling, likely by loosening the cell wall via oxidation, and (2) plasma-generated reactive species depolarize the spore membrane by increasing the activation of membrane Ca^2+^ ion influx systems, which may elevate intracellular Ca^2+^ levels and activate signaling pathways (e.g. MAPK signaling) that enhance the expression of germination-associated genes. In this study, plasma-treated spores swelled a little faster than the control spores. The enhanced swelling of plasma-treated spores may be the result of cell wall loosening via plasma-induced oxidation, a notion partially supported by our observations. Plasma treatment has been shown to accelerate the expression of a thaumatin-like gene encoding a hydrolytic enzyme that degrades cell wall components, loosens cell walls, and promotes spore swelling^46^.

Our study demonstrated that plasma treatment depolarizes spore membranes and elevates intracellular Ca^2+^ levels, likely via the activation of a Ca^2+^ membrane channel (Fig. 9). Membrane depolarization indicates the activation of ion influx systems in the cell membrane, such as the Ca^2+^ influx system which can be activated in response to oxidative stress^47^. Consequently, the intracellular Ca^2+^ level is elevated, triggering many cellular processes such as germination. Plasma appears to cause membrane oxidation which, in turn, may activate the Ca^2+^ ion influx system. Moderate membrane oxidation may stimulate ion channels promoting membrane transport^48^. Although experimental evidence for membrane lipid oxidation was not provided in this study, our data clearly indicated that plasma triggered membrane depolarization and elevated Ca^2+^ influx. Ca^2+^ membrane channel proteins activated following plasma treatment were not identified in the current study. However, a recent study demonstrates that He-plasma irradiated HEPES-buffered saline solution elicits an increase in Ca^2+^ level in fibroblast cell through activating transient receptor potential (TRP) channels^49^. In addition, several voltage gated Ca^2+^ channels are reportedly involved in the regulation of fungal cell differentiation and virulence^50,51^. These channel proteins may be activated by plasma, thus further investigation of this process is required. Another channel protein, aquaporin, may also be a candidate for stimulation by plasma since its activation may enhance water uptake inside spore cells, leading to spore swelling (Fig. 9). Recently, aquaporin was proposed to be a passage for plasma generated H_2_O_2_^52^. Our findings suggest an alternative possibility, whereby aquaporin may be stimulated by plasma-generated reactive species to enhance water uptake into the cell. Interestingly, our data indicated that compared to H_2_O_2_, plasma-generated NO enhanced membrane depolarization more efficiently. This may be due to the NO concentration in plasma-treated PBS and PDB being much higher than that of H_2_O_2_. However, the relative importance of H_2_O_2_ itself in plasma activation cannot be excluded.

Increased levels of intracellular Ca^2+^ may act as a secondary messenger triggering signal transduction, leading to the expression of genes regulating cell differentiation and stress responses^53^. Our data demonstrate that plasma-induced increases in intracellular Ca^2+^ levels may enhance the transcription of several genes known to be highly expressed during the early stages of *A*. *oryzae* germination, possibly by activating the MAPK, mpkA (Slt2-like). Crosstalk has often been demonstrated between calcium and MAPK signaling pathways in fungi^53^. We found that mpkA activation, as well as the expression of several germination-associated genes, including AO090005001280 and AO090003000685, was enhanced in response to plasma treatment. As stated previously, AO090005001280, which encodes a thaumatin-like protein, is known to be involved in spore swelling (isotropic growth) during germination. AO090003000685 is a transcription factor, AtfA, which enhances spore germination by regulating the expression of genes involved in *A*. *oryzae* germination^41^. MpkA (yeast Slt2-like MAP kinase) activated by plasma may increase the expression of these genes.

In conclusion, poor and unstable culture growth following isolation is a technical barrier to the efficient application of fungi to fermentation. Our study demonstrates that non-thermal atmospheric pressure plasma could be used to overcome this barrier. Plasma enhances fungal spore germination, which is the first, crucial step in fungal colonization, by activating spore swelling and Ca^2+^ influx. Although further studies on the effects of plasma and their underlying mechanisms are still needed, our findings demonstrate that plasma could facilitate the high-density fungal colonization required for efficient fermentation.

## Methods

### Fungal strains and culture conditions

The *A*. *oryzae* (KACC47488) strain used in this study was obtained from the Korean Agriculture Type Collection in the National Agrobiodiversity Center (Republic of Korea) and maintained on potato dextrose agar (PDA) medium (MB cell, Los Angeles, CA, USA) at 30 °C in the dark. For experimental purposes, the fungus was propagated on PDA or in PDB (Potato Dextrose Broth) at 30 °C.

### Atmospheric pressure non-thermal plasma device

A micro Dielectric Barrier Discharge (DBD) plasma unit equipped with a burst pulse type high voltage inverter was used in this study (Fig. 1). The structure of the microelectrodes and organization of the dielectric and Al_2_O_3_ layers in the device were as described previously^35^. A burst pulse type power system with on and off pulse times of 28.8 and 160 ms, respectively, was used in the plasma device. To generate plasma, nitrogen (N_2_) gas was injected into the device at a flow rate of 1 L per min, an input voltage of 1.2 kV, and a current of 50–63 mA.

### Treatment of fungal spores with plasma and spore germination analysis

*A*. *oryzae* spores were collected from 1-week-old culture plates. Approximately 15 mL of sterile phosphate buffered saline (PBS) was added to the plate and fungal material was scraped using an L-Spreader. The scraped suspension was filtered through four layers of sterile Miracloth (Calbiochem, Darmstadt, Germany) and centrifuged at 3,134 × *g* for 5 min. The liquid portion was discarded and the spore pellet was resuspended in PBS or PDB (Potato Dextrose Broth) at a concentration to 10^7^ spores per mL. One mL of suspension (10^7^ fungal spores) was placed on a petri dish (90 mm in diameter) and exposed to plasma at a distance of 10 mm for the indicated time periods. Spores exposed only to N_2_ gas were used as the control. Following treatment, the spore suspension was serially diluted using PBS or PDB solution and 100 µL of diluted suspension was spread onto PDA plates. The plates were incubated at 30 °C in the dark for 2 days and the number of colonies (germinated spores) was counted. The relative spore germination percentage compared with that of the control (gas-treated only) was calculated as follows: relative germination (%) = (the number of germinated spores after plasma or gas treatment/the number of germinated spores after gas treatment) × 100. The average germination percentage was calculated from three replicate plates per experiment and the experiment was repeated eight times.

To analyze spore size and examine spore swelling, spores (10^7^) treated with N_2_ gas (control) or plasma were incubated for 1, 2, and 3 h and applied to a BD FACSVerse™ flow cytometer (Becton Dickinson, San Jose, CA, USA). Forward scattered light (FSC) was measured using a 488 nm laser light and displayed as a single parameter histogram. The analysis was performed for two replicate experiments.

### Surface analysis by scanning electron microscope (SEM) and Fourier-transform infrared spectroscopy (FTIR)

The surface morphology of the fungal spores was analyzed using SEM following plasma treatment. Samples were prepared as described previously^35^. After mounted on carbon tape and coated with platinum, samples were examined under a scanning electron microscope (SEM) (JEOL, Tokyo, Japan).

Fungal spores in PBS and PDB were exposed to micro DBD plasma for 2 and 5 min, respectively, washed once with water, resuspended in 50 µL deionized water, and placed on a mounting plate and kept aside until the water evaporated. The dried spores were analyzed via FTIR as described previously^54^. Two replicate FTIR spectra measurements for each sample were averaged using Excel.

### Membrane fluidity and potential assay

To analyze fungal membrane fluidity and potential, *A*. *oryzae* spores (10^7^ spores) suspended in PBS or PDB were treated with N_2_ gas (control) and plasma for 2 min and 5 min, respectively. After treatment, the fluidity of the fungal cell membrane was analyzed using a Membrane Fluidity kit (Abcam, MA USA), following the manufacturer’s protocol. The treated spores were washed with PBS and suspended in 500 μL labeling solution (0.08% F-127, 30 µM pyrenedecanoic acid). Following incubation at 25 °C in the dark for 30 min, the spores were washed twice in PBS and resuspended in 1 mL of PBS. The spore suspensions were transferred into individual wells of a 96-well plate (Corning Incorporated, NY, USA) and their fluorescence was measured at 310 nm (ex.), 420 nm (em.), or 485 nm (em.) using a Synergy HTX Multi-Mode Reader (BioTek Instruments, VT, USA).

In order to analyze membrane potential, the treated spores were incubated in 1 mL of PBS containing 50 μg of bis-(1,3-dibutylbarbituric acid) trimethine oxonol (DiBAC4(3); 490 ex./516 em. nm; Molecular Probes, Eugene, OR, USA) for 1 h at 4 °C in the dark. After incubation, the level of fluorescence was analyzed using a BD FACSVerse™ flow cytometer (becton Dickinson, San Jose, CA, USA).

### Intracellular calcium level assay

A fluorescent calcium indicator (Fluo-3, AM; 506 ex./526 em. nm; Molecular Probes, Eugene, OR, USA) was used to stain intracellular calcium in *A*. *oryzae* spores. Fungal spores (10^7^ spores) were treated with N_2_ gas (control) and plasma for 2 and 5 min in PBS and PDB solutions, respectively, and incubated at 30 °C for 0, 2, and 4 h. Fungal spores were then washed with PBS and incubated in 10 mM Fluo-3 in PBS at 30 °C for 20 min. The spores were washed with PBS again and fluorescence was detected via a confocal laser scanning microscope (Olympus Corporation, Tokyo, Japan).

### Quantitative PCR analysis

To measure the mRNA levels of genes associated with spore germination, spores in PBS or PDB media were treated with N_2_ gas (control) and plasma for 2 min and 5 min, respectively, and cultured at 30 °C with shaking. Fungal samples were harvested at specific time points during incubation, washed twice with PBS, and stored at −80 °C. Total RNA was extracted using a TaKaRa RNAiso Plus kit (TaKaRa Bio, Tokyo, Japan) according to the manufacturer’s protocol. The RNA concentration was measured using a nanodrop (Biotek Instruments, VT, USA). The same amount of RNA (0.5 µg) was used to synthesize cDNA using the ReverTra Ace qPCR RT Master Mix with gDNA remover following the manufacturer’s protocol (Toyobo, Osaka Japan). Ten putative genes related to *A*. *oryzae* spore germination were amplified and quantified at every thermal cycle using iQ SYBR Green Supermix (BioRad, Hercules, CA, USA) and a CFX96TM real time RT-PCR system (Bio-Rad). The thermal cycling conditions were as follows: 95 °C for 3 min; 40 cycles at 95 °C for 10 sec; and 60 °C for 30 sec. Relative mRNA levels were expressed as a ratio compared to that of a reference gene (18S ribosomal RNA). Cycle threshold (Ct) values were determined and the difference in Ct values between plasma- and N_2_ gas (control)-treated samples was used to calculate the relative target gene expression level as follows: Ratio = (2)^∆Ct target (control-sample)^/(2)^∆Ct reference (control-sample)^^55^. The sequences of all primers used are listed in Table 2. Three replicate measurements were averaged in each experiment and the experiment was repeated three times.

### MAP kinase phosphorylation analysis

Fungal spores (10^7^ spores) in PBS and PDB were treated with N_2_ gas (control) and plasma for 2 and 5 min, respectively, and then incubated at 30 °C for 4 h. Spores were harvested, washed with PBS, and stored at −80 °C. To extract total proteins, fungal cells were ground in liquid nitrogen and the ground powder was transferred into a 2 mL microfuge tube containing 1 mL of chilled 3 mM phenylmethylsulfonyl fluoride (PMSF) in 95% ethanol with approximately 0.2 g of glass beads (0.5 mm diameter). Tubes were vortexed three times (60 sec each time), with 60 sec rests on ice in between. Extracts were chilled at −20 °C for at least 16 h. Samples were then centrifuged at 14,000 rpm for 10 min at 4 °C. The supernatant was removed and the pellet (containing proteins) was dried in a vacuum dryer for 30 min. To extract proteins, the pellet was reconstituted in 250 μL of 1% SDS, heated at 85 °C for 5 min, and centrifuged at 14,000 rpm for 5 min at room temperature. The protein supernatant was transferred to a new microfuge tube, the extraction steps were repeated once, and the supernatants were combined. The extraction steps were repeated once again with the combined supernatant to remove residual cellular debris. Protein concentration was determined using the Bradford protein assay (Bio-Rad, Hercules, CA, USA).

Phosphorylation of the three MAPKs was examined via western blot analysis. An equal amount of extracted total protein (30 μg) was subjected to 12% SDS polyacrylamide gel electrophoresis and the gel was blotted onto nitrocellulose membranes (0.45 μM; Bio-Rad, Hercules, CA, USA). Blotted membranes were then blocked in TBST buffer (10 mM Tris-Cl, pH 8.0, 150 mM NaCl, and 0.05% Tween 20) containing 5% milk for 1 h at room temperature and incubated with primary antibodies (anti-p38, anti-p44/42, anti-phospho-p38, and anti-phospho-p44/42; Cell Signaling Technology, Danvers, MA) in the blocking solution (1:200 dilution) at 4 °C overnight. After incubation, the membranes were washed three times with TBST buffer (5 min each) and incubated with secondary antibodies (anti-rabbit IgG, HRP-linked antibody, 1:7500 dilution; Cell Signaling Technology, Danvers, MA) for 1 h at room temperature. The membranes were washed three times with TBST, treated with Clarity Western ECL Substrate (BioRad, Hercules, CA, USA), and chemiluminescence was detected using a ChemiDoc^TM^ MP Imaging System (BioRad, Hercules, CA, USA).

### Measurement of H_2_O_2_ and NO level in the background media

To analyze H_2_O_2_ and NO levels in the background media, 1 mL of PBS or PDB were treated with N_2_ gas (control) and plasma for the indicated time periods. The H_2_O_2_ concentration was analyzed using an Amplex™ Red Hydrogen Peroxide/Peroxidase Assay Kit (Molecular Probes, Eugene, OR, USA) and the NO level was analyzed using a QuantiChromTM Nitric Oxide Assay Kit (BioAssay Systems, Hayward, CA, USA), according to the manufacturer’s protocols.

### Statistical analysis

All values are expressed as the mean ± standard deviation of three replicate measurements from at least three repeated experiments. Statistical analysis of the data was performed using Student’s *t*-tests to determine the significance of differences between treatments. Statistical significance was set at *p* < 0.05 (*) or *p* < 0.01 (**).

## Supplementary information

Supplementary figures
