# Supplementary material for: Aspergillus oryzae spore germination is enhanced by non-thermal atmospheric pressure plasma
Source: Sci Rep. 2019 Aug 1;9:11184. doi: 10.1038/s41598-019-47705-4 (PMC6673704; doi:10.1038/s41598-019-47705-4)
Supplement: Supplementary file 1 — Supplementary figures [file 41598_2019_47705_MOESM1_ESM.pdf]

Supplementary Information

Aspergillus oryzae spore germination is enhanced by non-thermal atmospheric pressure plasma

Mayura Veerana[1,2], Jun-Sup Lim[3], Eun-Ha Choi[1,2,3], Gyungsoon Park[1,2,3]*

[1] Plasma Bioscience Research Center, Kwangwoon University, Seoul, 01897, Korea

[2] Department of Plasma Bioscience and Display, Kwangwoon University, Seoul, 01897, Korea

[3] Department of Electrical and Biological Physics, Kwangwoon University, Seoul, 01897, Korea

[*] Corresponding author

Phone: +82-2-940-8324

Fax: +82-2-940-5664

Email: gyungp@kw.ac.kr

Supplementary Methods

Monitoring the spore germination in *A. oryzae* wild type over time

A. oryzae spores were collected from 1-week-old culture plates. About 15 ml sterile PBS (Phosphate Buffered Saline) was added into the plate, and the fungal material was scraped using L-Spreader. The scraped suspension was filtered through 4 layers of sterile Miracloth (Calbiochem, Darmstardt, Germany), and then the filtered suspension was centrifuged at 3,134 *g* for 5 min. After liquid part was discarded, the spore pellet was resuspended in PDB (Potato Dextrose Broth), adjusting the concentration to 10^7 spores per mL. The spore suspension was incubated at 30 °C with shaking (180 rpm). At every indicated time, about 10 µl suspension was mounted on the slide glass and observed under the Leica ICC50 E microscope (Leica Microsystems, Heerbrugg, Switzerland).

Measurement of the concentration of nitric oxide in sodium nitroprusside (SNP) solution

Sodium nitroprusside (SNP; Sigma-Aldrich, St. Louis, MO, USA) was dissolved in deionized water at different concentrations. Nitric oxide (NO) released in SNP solutions of different concentrations was quantitated using QuantiChromTM Nitric Oxide Assay Kit, (BioAssay Systems, Hayward, CA, USA), following the manufacturer's protocol.

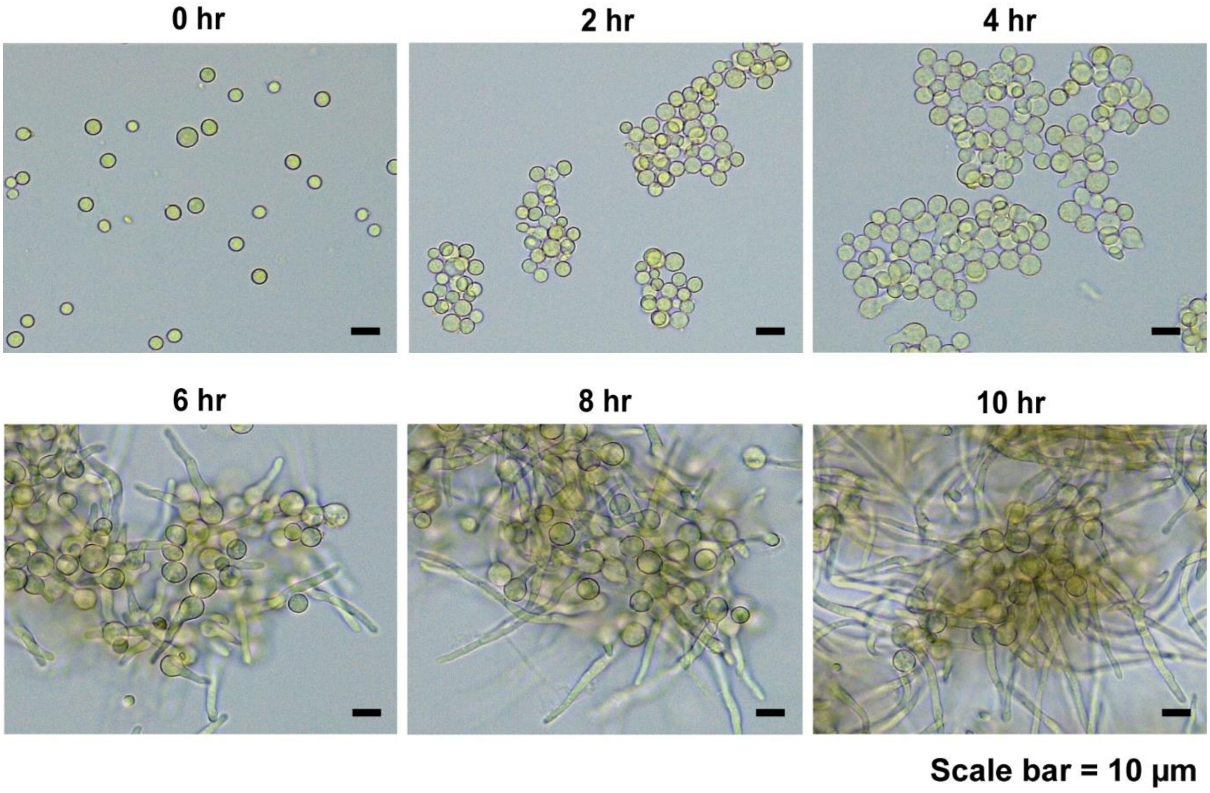

Supplementary Figure S1. **Spore germination in *A. oryzae*.**

Germination of spores of *A. oryzae* wild type was observed under the microscope during incubation in PDB. Experimental procedure was described in the supplementary methods.

1 h	2 h	3 h

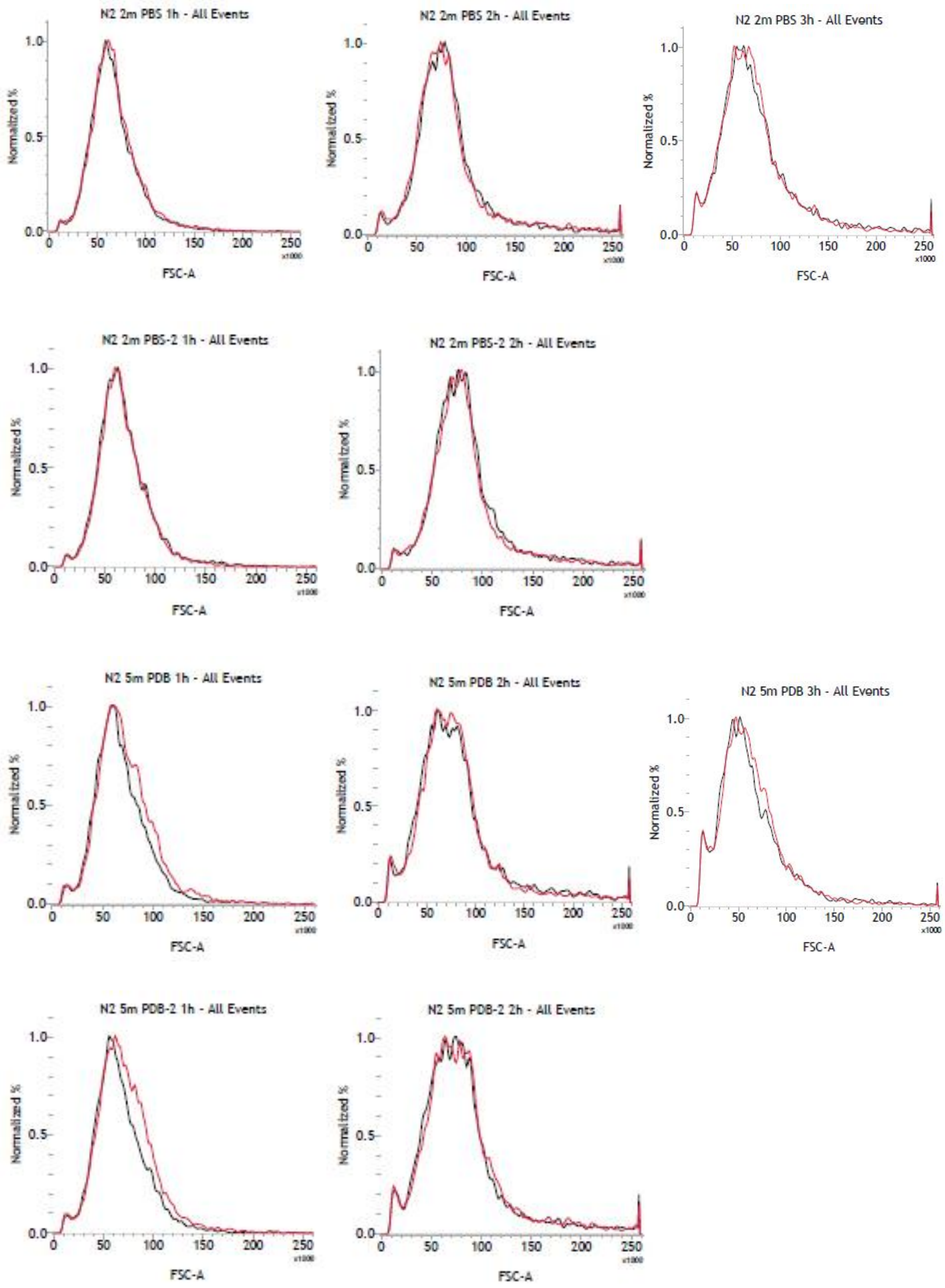
N2 2m PBS 1h - All Events
N2 2m PBS 2h - All Events
N2 2m PBS 3h - All Events
N2 2m PBS-2 1h - All Events
N2 2m PBS-2 2h - All Events
N2 5m PDB 1h - All Events
N2 5m PDB 2h - All Events
N2 5m PDB 3h - All Events
N2 5m PDB-2 1h - All Events
N2 5m PDB-2 2h - All Events
Normalized %
FSC-A
x1000

Supplementary Figure S2. FACS analysis for estimating the size of spores after plasma treatment.

Spores (10^7 spores) in PBS and PDB were treated with micro DBD plasma for 2 min and 5 min, respectively. Black and red lines represent control (N_2 gas only) and plasma treatment. Experiment was repeated once.

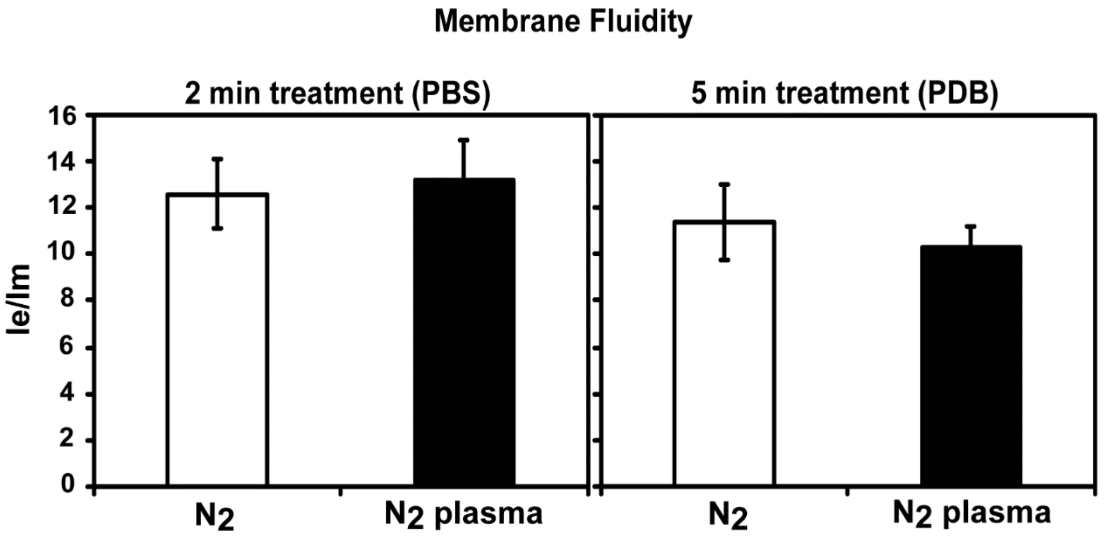

Supplementary Figure S3. **The fluidity of fungal plasma membrane after plasma treatment.**

Fungal spores were treated with nitrogen gas and plasma for 2 min in PBS and 5 min in PDB solutions and then stained with lipophilic pyrene probes to examine the fluidity of the plasma membrane. Each value was the average ± standard deviation of 6 replicates.

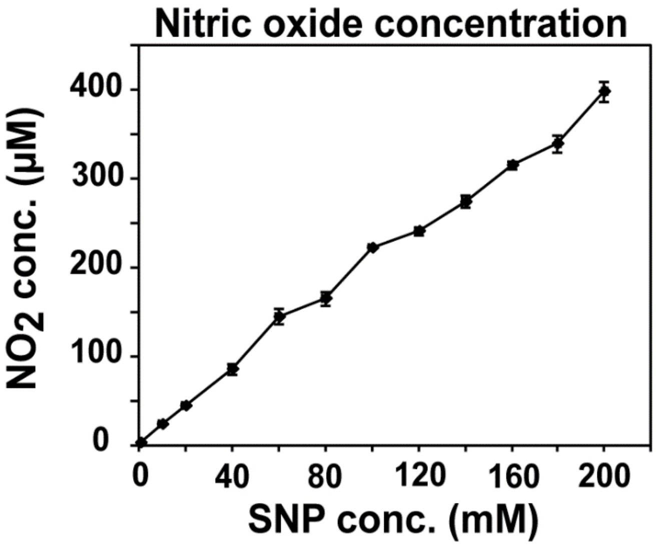

Supplementary Figure S4. **Concentration of NO in SNP solutions.**

NO released in different concentration of SNP solution. Assay for NO concentration was described in the supplementary methods. All measurements were performed in 3 replicates.

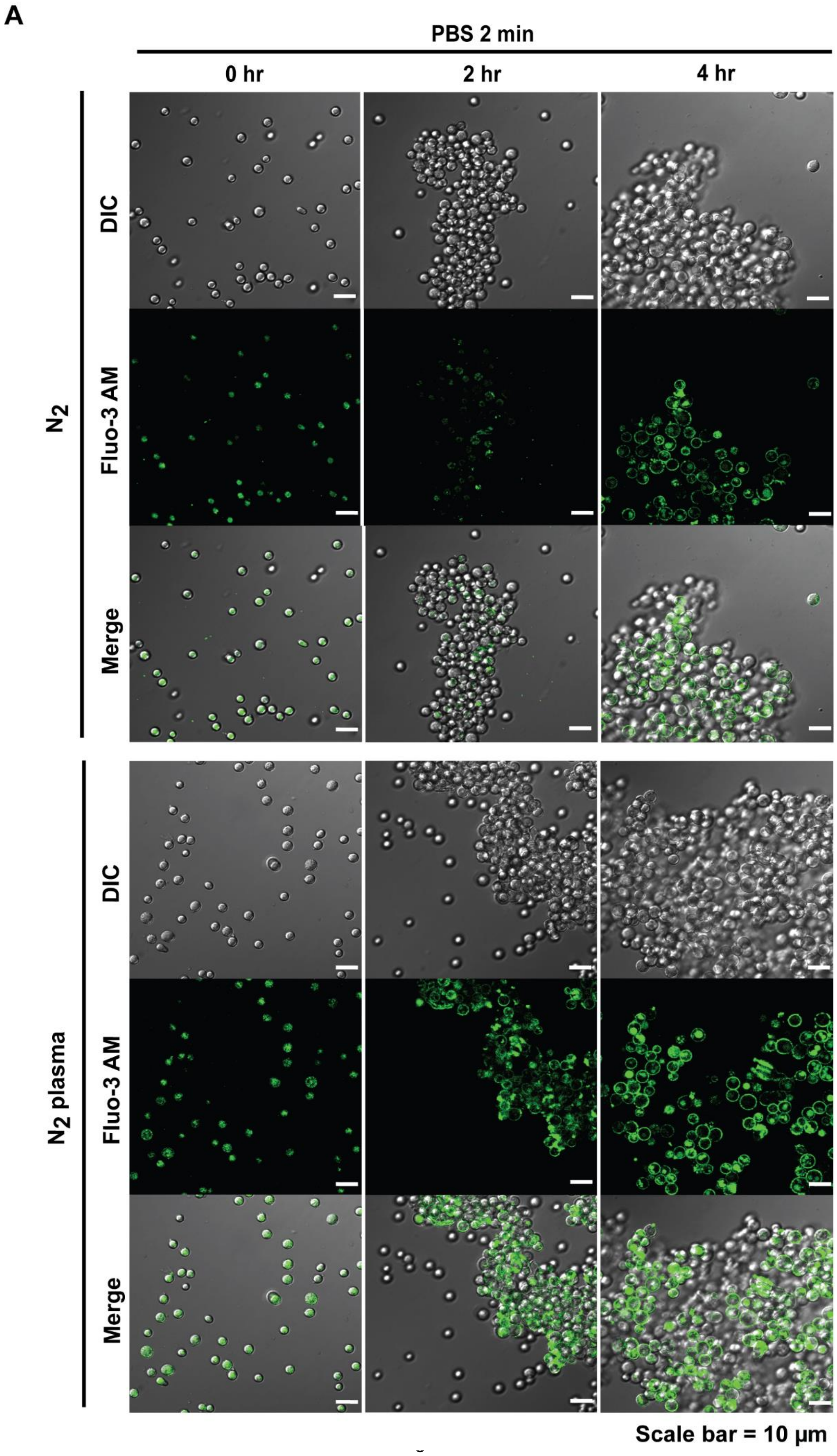
A
PBS 2 min
0 hr
2 hr
4 hr
N2
DIC
Fluo-3 AM
Merge
N2 plasma
DIC
Fluo-3 AM
Merge
Scale bar = 10 μm

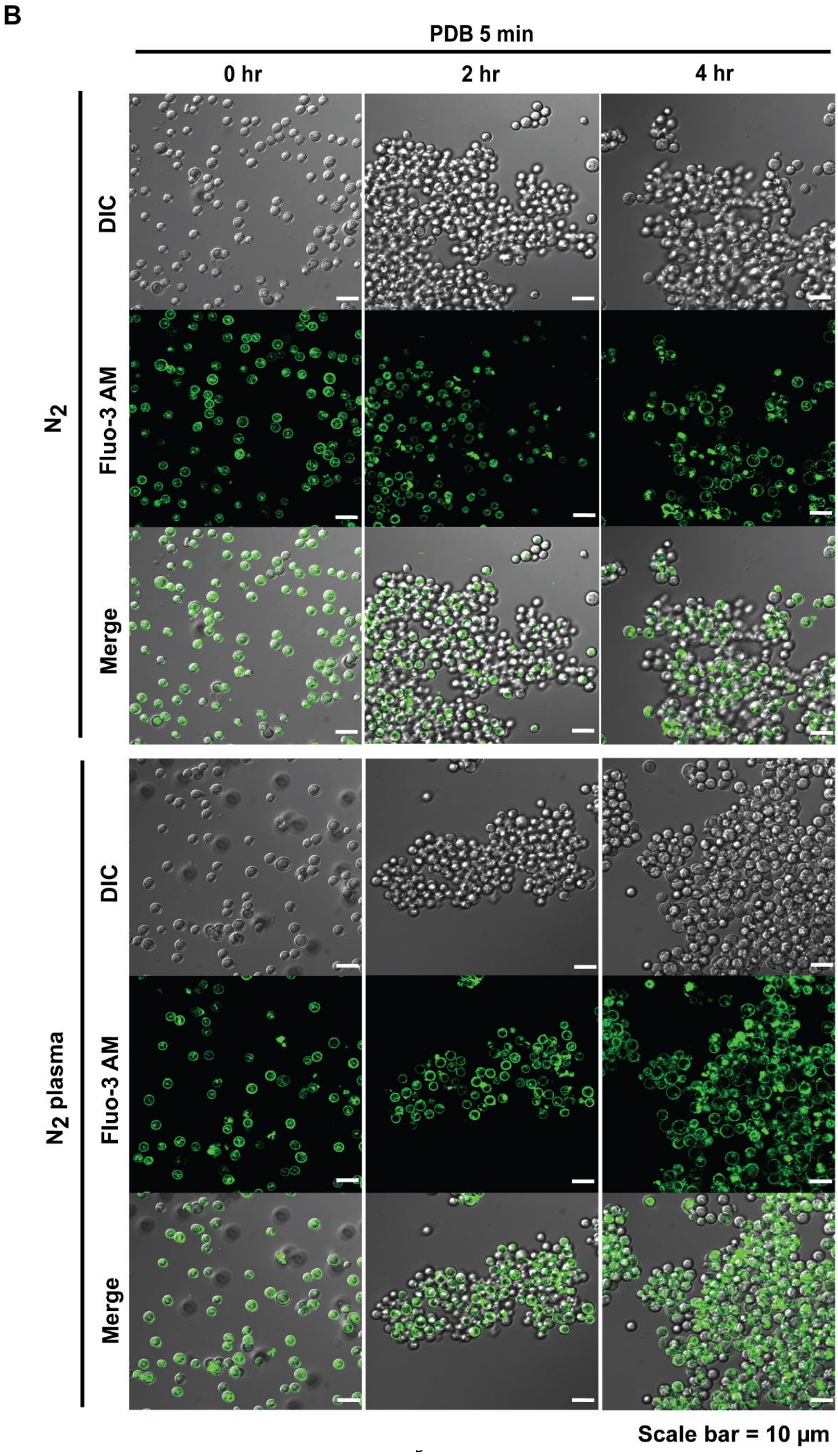
B
PDB 5 min
0 hr
2 hr
4 hr
N_2
DIC
Fluo-3 AM
Merge
N_2 plasma
DIC
Fluo-3 AM
Merge
Scale bar = 10 μm

Supplementary Figure S5. **Intracellular Ca^{2+} level in fungal spores after plasma treatment.**

Fluo-3 AM probe was used to detect intracellular calcium in fungal spores. **A.** Intracellular Ca^{2+} in fungal spores after plasma treatment in PBS for 2 min. **B.** Intracellular Ca^{2+} in fungal spores after plasma treatment in PDB for 5 min. DIC; Differential Interference Contrast, Fluo-3 AM; fluorescence, Merge; Combined image of DIC and fluorescence

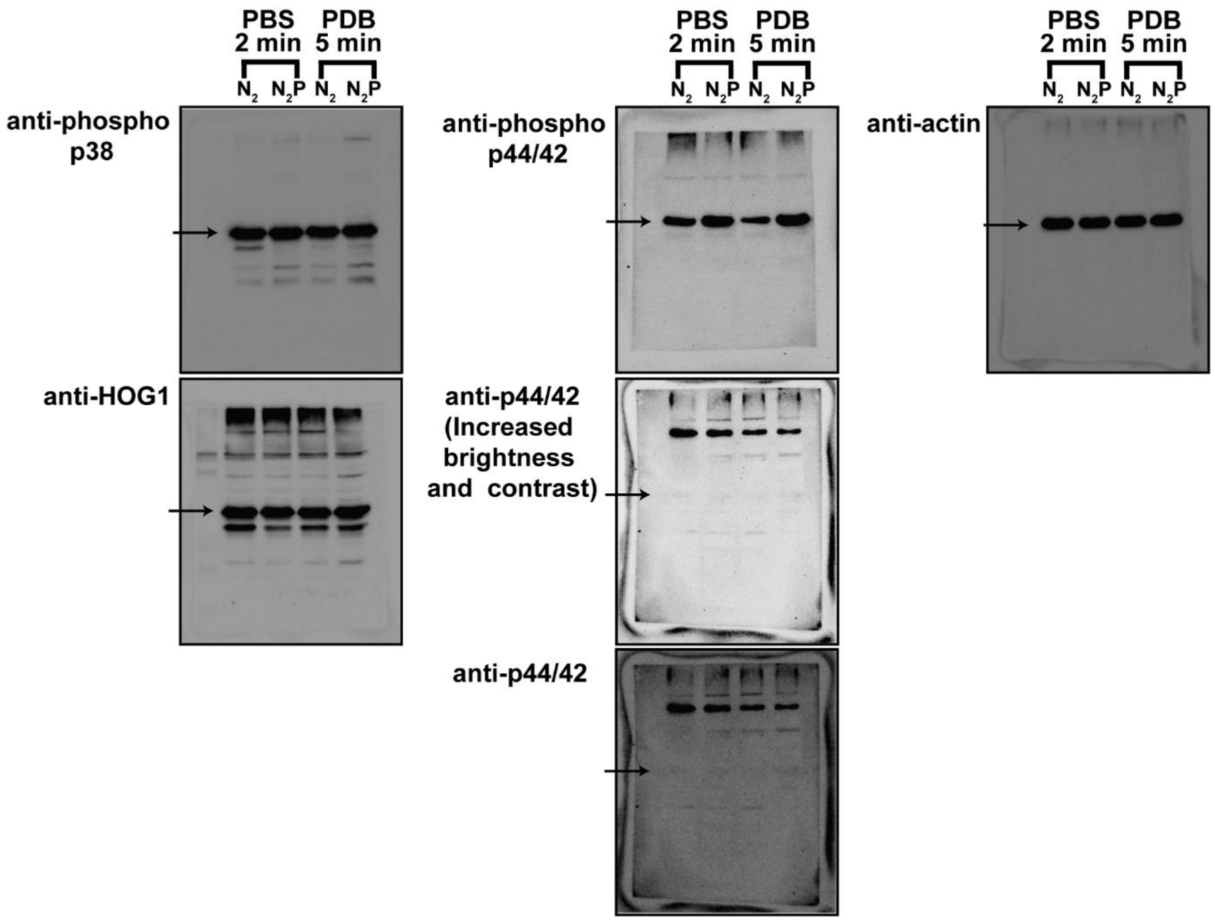

Supplementary Figure S6. **Western blot results for the phosphorylation of MAP kinases following plasma treatment.**

Total protein (30 μg) extracted from fungal spores incubated for 4 h following plasma treatment (2 min in PBS and 5 min in PDB) was subjected to SDS polyacrylamide gel electrophoresis. N_2; treatment with only N_2 gas, N_2P; treatment with N2 plasma. Arrows indicate protein bands corresponding to each MAP kinase (hogA or mpkA) with or without phosphorylation, and β-actin (reference protein). The phosphorylation and protein levels of each MAP kinase were detected using following antibodies: hogA (expected molecular weight 41.8 kDa); anti-phospho p38 (phosphorylation) and anti-Hog1 (protein), mpkA (expected molecular weight 47.8 kDa); anti-phospho p44/42 (phosphorylation) and anti-p44/42 (protein).

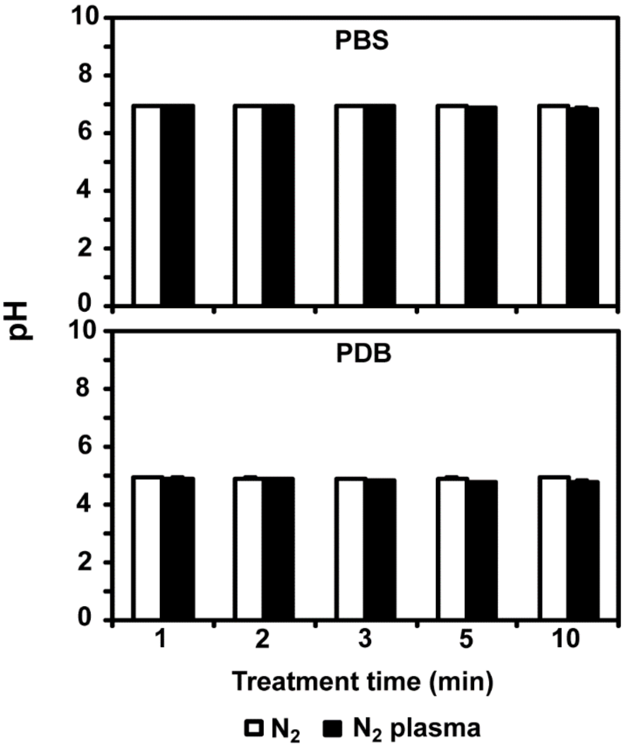

Supplementary Figure S7. pH of PBS and PDB solutions after plasma treatment for the indicated time.

Each value is the average of 3 replicate measurements.
